# Character Strengths Predict an Increase in Mental Health and Subjective Well-Being Over a One-Month Period During the COVID-19 Pandemic Lockdown

**DOI:** 10.3389/fpsyg.2020.584567

**Published:** 2020-10-21

**Authors:** María Luisa Martínez-Martí, Cecilia Inés Theirs, David Pascual, Guido Corradi

**Affiliations:** Faculty of Health and Education, Camilo José Cela University, Madrid, Spain

**Keywords:** character strengths, COVID-19, pandemic, mental health, subjective well-being, resilience, longitudinal design

## Abstract

This study examines whether character strengths predict resilience (operationalized as stable or higher mental health and subjective well-being despite an adverse event) over a period of approximately 1 month during the COVID-19 pandemic lockdown in Spain. Using a longitudinal design, participants (*N* = 348 adults) completed online measures of sociodemographic data, information regarding their situation in relation to the COVID-19, character strengths, general mental health, life satisfaction, positive affect and negative affect. All variables were measured at Time 1 and Time 2, except for sociodemographic and most COVID-related information (Time 1 only). Time 1 data collection was conducted between March 21, 2020 and April 2, 2020, i.e., approximately the second week of lockdown in Spain. Time 2 data collection was conducted between April 24, 2020 and May 18, 2020, after the Spanish government announced its intention to progressively release the lockdown. A principal component analysis of character strengths was conducted. Five character strength factors were extracted: *fortitude*, *goodness*, *intellectual*, *interpersonal*, and *restraint*. Factor structures at Times 1 and 2 were highly consistent. All character strength factors at Time 1 correlated positively with life satisfaction and positive affect, and negatively with negative affect and mental health at T2 (higher scores in the mental health measure indicate poorer mental health). *Fortitude strengths* showed the highest correlations. We conducted a series of regression analyses with strength factors at Time 1 as predictors, and mental health, life satisfaction, and positive and negative affect as dependent variables, controlling for their baseline levels. To test the directionality of the relationship between strengths and the dependent variables, all analyses were reversed. All character strength factors predicted an increase in mental health. They also predicted positive affect, with the exception of *strengths of restraint*. *Fortitude*, *intellectual*, and *interpersonal strengths* predicted an increase in life satisfaction. Finally, *fortitude strengths*, *interpersonal strengths*, and *strengths of restraint*, predicted a decrease in negative affect. None of the reversed analyses yielded significant effects. Limitations, implications, and possible character strengths-based interventions aimed at promoting mental health in the COVID-19 pandemic are discussed.

## Introduction

The COVID-19 pandemic has caused an international public health emergency with multiple economic and social consequences. The disease was declared a pandemic by the World Health Organization on March 11, 2020, with more than 118000 confirmed cases worldwide and a death toll of 4291. Currently, the pandemic affects 114 countries (World Health Organization [WHO], 2020). In Spain, the government announced a nationwide lockdown on March 14, 2020 (Real Decreto [RD] 463, 2020 of March 14), with 7641 confirmed cases and 141 deaths (EpData, 2020). The Spanish population was confined at home with limited exceptions for essential supplies, critical business needs, or urgent medical assistance. On March 29, 2020, even stricter lockdown measures were announced, and all non-essential workers were ordered to remain at home for the following 2 weeks (Real Decreto-Ley [RDL] 10, 2020 of March 29). These new measures were imposed to avoid a collapse of the already-saturated hospital network. Between March 29, 2020, and April 11, 2020, the infection curve in Spain peaked, and the number of new cases and deaths started decreasing (EpData, 2020. On April 28, 2020, the government announced a de-escalation plan composed of four phases (0–3), basing the transition from one phase to another on public health indicators (Ministerio de Sanidad, Consumo y Bienestar Social [MSCBS], 2020a). As phase 0 of this de-escalation plan, on May 2, 2020, restrictions were eased and the population was allowed to go out for short walks or do individual sports, and on May 11, half of the Spanish population entered phase 1, which included the opening of outdoor bars at 50% capacity, small shops, and places of worship at one-third of their capacity. COVID-19’s greater impact on regions such as Madrid and Barcelona blocked their transition to phase 1 until May 25, 2020 (Ministerio de Sanidad, Consumo y Bienestar Social [MSCBS], 2020b).

Containment measures for diseases such as quarantine and isolation can be traumatic for a percentage of the population. In a United States study on the effects of the H1N1 pandemic, Sprang and Silman (2013) found that 25% of quarantined or isolated adults presented post-traumatic stress disorder (PTSD). These percentages are similar to those observed in studies regarding the SARS pandemic and to those found in other potentially traumatic events such as natural disasters and terrorism (Hawryluck et al., 2004). There is abundant literature on the negative effects of traumatic events and disasters on humans (Norris et al., 2002). However, is it possible that some people have coped with the current pandemic in a healthy way despite the adverse circumstances? If this is so, what factors were responsible for this?

An emerging field of research has begun to show that a large percentage of the general population is usually resilient, i.e., capable of maintaining healthy levels of subjective and psychological well-being despite adverse circumstances (Bonanno, 2004). In his compelling article, Bonanno (2004) cited several such studies. For example, Zisook et al. (1997) observed that approximately half of a sample of conjugally bereaved adults did not show even mild depression (i.e., fewer than two items from the DSM–IV symptom list) after the loss. In another study of resilience to loss, 46% of the sample had low levels of depression, both prior to the loss and through 18 months of bereavement, and had relatively little grief during bereavement (Bonanno et al., 2002). Additionally, studies on violent and life-threatening events showed even higher percentages of resilient individuals. For example, among hospitalized survivors of motor vehicle accidents (Bryant et al., 2000), 79% of the sample did not meet criteria for PTSD. In another study, 62.5% of Gulf War veterans had no psychological distress when examined within 1 year of their return to the United States (Sutker et al., 1995). Other studies on the psychological effects of traumatic situations such as the terrorist attacks that occurred in 2001 (New York), 2004 (Madrid), or 2005 (London), have shown that most people in the general population exposed to these traumatic events did not develop a psychological disorder related to this situation (Rubin et al., 2005; Bonanno et al., 2006; Matt and Vázquez, 2008; Vázquez et al., 2008). For example, Bonanno et al. (2006) observed resilience in 65.1% of a sample of New York residents after the 9/11 terrorist attack, even though many participants had a high exposure to the event. In fact, in the days immediately following the terrorist attacks, most people experienced more positive than negative emotions (Smith et al., 2001), and Fredrickson et al. (2003) showed that experiencing positive emotions, such as gratitude, love, or interest, in the days following the 9/11 terrorist attack, mediated the relationship between pre-attack resilience and decreased depression, as well as increased growth in psychological resources, after the attack.

In the specific case of pandemics, studies are considerably scarcer and are mainly based on the assessment of clinical symptoms. Some studies focused on certain positive aspects, although they do not usually evaluate measures such as well-being. An example of this is the study carried out in Hong Kong on the effects of the SARS epidemic in 2003, in which greater social/family support, awareness of one’s own mental health and time spent on healthy practices such as rest, relaxation or physical exercise were observed (Lau et al., 2006). However, as some authors claim (e.g., Vázquez et al., 2008), to adequately measure resilience, it is not enough to measure the absence of clinically significant symptoms, but rather to evaluate aspects such as people’s daily functioning and their adaptive reaction to adversity, the learning experienced from the experience, or measures of well-being, such as positive emotions.

Recently, within the field of positive psychology, research has begun on the role of character strengths in coping with adverse situations. Peterson and Seligman (2004) defined character strengths as positive, morally valued personality traits. They are traits in the sense of being individual differences with a certain degree of temporal stability and generality, but they are not necessarily fixed or based on immutable biogenetic characteristics. Peterson and Seligman (2004) proposed a classification of 24 character strengths that are assigned to one of six universal virtues (see Table 1). Virtues are the central characteristics of character, valued by religious thinkers and philosophers, while character strengths are the psychological routes in which the virtues are manifested.

**TABLE 1:** VIA Classification of six virtues and 24 character strengths (Peterson and Seligman, 2004) and the respective strengths factors in the present study in brackets.

Virtue I. Wisdom and knowledge: cognitive strengths that entail the acquisition and use of knowledge
(1) Creativity: thinking of novel and productive ways to do things (*Intellectual strengths*)
(2) Curiosity: taking an interest in all of ongoing experience (*Intellectual strengths*)
(3) Open-mindedness: thinking things through and examining them from all sides (*Intellectual strengths*)
(4) Love of learning: mastering new skills, topics, and bodies of knowledge (*Intellectual strengths*)
(5) Perspective: being able to provide wise counsel to others (*Intellectual strengths*)
Virtue II. Courage: emotional strengths that involve the exercise of will to accomplish goals in the face of opposition, external or internal.
(6) Bravery: not shrinking from threat, challenge, difficulty, or pain (*Fortitude strengths*)
(7) Persistence: finishing what one starts (*Fortitude strengths*)
(8) Integrity: speaking the truth and presenting oneself in a genuine way (*Goodness strengths*)
(9) Vitality: approaching life with excitement and energy (*Fortitude strengths*)
Virtue III. Humanity: interpersonal strengths that involve “tending and befriending” others.
(10) Love: valuing close relations with others (*Goodness strengths*)
(11) Kindness: doing favors and good deeds for others (*Goodness strengths*)
(12) Social intelligence: being aware of the motives and feelings of self and others (*Interpersonal strengths*)
Virtue IV. Justice: civic strengths that underlie healthy community life.
(13) Citizenship: working well as member of a group or team (*Interpersonal strengths*)
(14) Fairness: treating all people the same according to notions of fairness and justice (*Strengths of restraint*)
(15) Leadership: organizing group activities and seeing that they happen (*Fortitude strengths*)
Virtue V. Temperance: strengths that protect against excess.
(16) Forgiveness and Mercy: forgiving those who have done wrong (*Goodness strengths*)
(17) Humility and Modesty: letting one’s accomplishments speak for themselves (*Strengths of restraint*)
(18) Prudence: being careful about one’s choices; not saying or doing things that might later be regretted (*Strengths of restraint*)
(19) Self-regulation: regulating what one feels and does (*Strengths of restraint*)
Virtue VI. Transcendence: strengths that forge connections to the larger universe and provide meaning.
(20) Appreciation of beauty and excellence: noticing and appreciating beauty, excellence, and/or skilled performance in all domains of life (*Interpersonal strengths*)
(21) Gratitude: being aware of and thankful for the good things that happen (*Goodness strengths*)
(22) Hope: expecting the best and working to achieve it (*Fortitude strengths*)
(23) Humor: liking to laugh and joke; bringing smiles to other people (*Interpersonal strengths*)
(24) Spirituality: having coherent beliefs about the higher purpose and meaning of life (*Fortitude strengths*)

*Source Martínez-Martí and Ruch (2017). VIA, values in action.*

There is initial evidence on the relationship between character strengths and resilience. Martínez-Martí and Ruch (2017) observed that all character strength factors (derived empirically using a principal component analysis), except for *theological strengths* (that included spirituality and gratitude), yielded significant positive correlations with resilience. Moreover, character strengths were able to explain a statistically significant percentage of the variance in resilience above other factors strongly related to resilience such as positive affect, self-efficacy, optimism, social support, self-esteem, satisfaction with life and sociodemographic variables (i.e., gender, age, and education). When including all variables in the model, *emotional strengths* (i.e., love, vitality, hope, humor, and social intelligence, in Martínez-Martí and Ruch, 2017 study) and *strengths of restraint* (i.e., persistence, self-regulation, prudence, open-mindedness, and perspective, in Martínez-Martí and Ruch, 2017) were significant positive predictors. All 24 character strengths showed positive significant correlations with resilience, except for humility (non-significant). The five individual character strengths that showed the highest correlations with resilience were, in decreasing order, hope, vitality, bravery, curiosity, and persistence (all above 0.50), while the five individual character strengths that showed the lowest correlations with resilience were, in ascending order, humility, prudence, spirituality, appreciation of beauty and excellence, and integrity (all below 0.27). Although this study showed initial evidence of the relationship between character strengths and resilience, it relied on a cross-sectional design, which precludes the possibility of making any inferences about causality.

Thus, this study aimed to examine the potential protective role of character strengths in this specific adverse situation. Specifically, we tested whether character strengths predicted an increase in mental health and subjective well-being (i.e., higher life satisfaction, higher positive affect and lower negative affect) over a period of approximately 1 month during the lockdown period in Spain. In order to test whether character strength factors predicted changes in mental health and subjective well-being over a period of approximately 1 month, we conducted a series of regression analyses on each character strength factor at Time 1 as a predictor, and mental health, life satisfaction, positive affect and negative affect at Time 2 as dependent variables. Since most research on character strengths has previously shown that the 24 character strengths are usually grouped into three (e.g., Shryack et al., 2010; McGrath, 2015), or five factors (e.g., Ruch et al., 2010; McGrath, 2014), character strength factors were derived empirically.

For this purpose, we conducted a principal component analysis, a procedure used previously in several studies (e.g., Ruch et al., 2010; Martínez-Martí and Ruch, 2017). Although computing character strength factors might involve a loss of information when studying character strengths, and the resulting factors might vary across studies, making it difficult to compare results across studies, it has the advantage of making the data analyses more manageable when studying the 24 character strengths altogether. When conducting the regression analyses, dependent variables’ baseline levels, i.e., at Time 1, were controlled. Moreover, to test the directionality of the relationship between character strengths and the dependent variables, i.e., to confirm that character strengths predicted mental health and subjective well-being over time, but not the other way round, all analyses were reversed. Specifically, the same regression analyses were performed but with mental health, life satisfaction, positive affect, and negative affect at Time 1 as predictors, and character strength factors at T2 as dependent variables, controlling for character strength factors at Time 1. We hypothesized that character strength factors would predict an increase (or at least stable levels) in mental health, life satisfaction and positive affect, and a decrease (or at least stable levels) in negative affect. Additionally, we expected that the reversed analyses would be non-significant. Moreover, we expected that the character strengths that have shown the highest correlations with resilience in previous studies (i.e., Martínez-Martí and Ruch, 2017), such as hope, vitality, bravery, curiosity, persistence, humor, perspective, and social intelligence, would be particularly important for mental health and subjective well-being in the current pandemic situation.

## Materials and Methods

### Participants

The sample consisted of 348 adults (262 women) with a mean age of *M* = 43.17 (*SD* = 11.29, range 19–82). All participants were residents in Spain. Most participants were Spanish (94.5%), followed by German (0.9%), Venezuelan and American (0.6% each), and other nationalities that represented 0.3% each, e.g., Argentinian, Italian, and Portuguese. Regarding education, 49.7% of the sample had a university degree or diploma, 28.2% had completed postgraduate studies, 11.8% had a PhD, 8.6% had graduated from secondary school, and 1.7% had graduated from primary school. Regarding their situation in relation to COVID-19 at Time 1, 85% of the sample had no symptoms of COVID-19, 0.9% had been infected, and 14.1% were unsure. Also, 56% of the sample did not know anyone close who had been infected, while 44% knew someone close who had been infected. Regarding the number of people living in the same household, 15.8% of the sample were living alone, 36.5% were living with another person, 24.4% were living with two other people, 15.8% with three other people, 6.6% with four other people, 0.3% with five other people, and 0.6% with six other people. Regarding their work situation, 58% of the sample were teleworking, 10.1% had continued going to their workplaces, 9.8% were temporally unemployed due to the lockdown, 2% were unemployed, and the remaining 20.1% reported “Other situation.” When participants were asked how many days per week they had left their houses since the lockdown had begun, 18.4% responded zero days, 31.6% of the sample went out 1 day per week, 21.3% 2 days, 8% 3 days, 3.2% 4 days, 6.9% 5 days, 0,9% 6 days, and 9.8% 7 days. The reasons reported for going out were mainly taking out the trash, grocery shopping, going to work, walking the dog or going to the doctor.

### Instruments

The Spanish translation of the *Character Strengths Rating Form* (*CSRF*; Ruch et al., 2014) was used to assess character strengths. The *CSRF* is a 24-item questionnaire with a 9-point Likert scale (from 1 = not like me at all through 9 = absolutely like me) that measures the 24 *VIA (Values in Action)*-character strengths, i.e., as per the model in Peterson and Seligman (2004). Each of the items on the *CSRF* describes one of the 24 character strengths, and participants indicate the degree to which the character strengths apply to them. Higher scores represent a higher endorsement of the strength. A sample item is: “Bravery (valor): Brave and courageous people do not shrink from threat, challenge, difficulty or pain. They speak up for their opinions and convictions even if there is opposition.” In the present study, character strengths were grouped into five factors: *fortitude strengths*, *goodness strengths*, *intellectual strengths*, *interpersonal strengths*, and *strengths of restraint*. The data reduction procedure is described in detail in the Data Reduction section. Cronbach’s alphas at Time 1 and Time 2 were, respectively, 0.80 and 0.82 (fortitude strengths), 0.79 and 0.81 (goodness strengths), 0.80 and 0.82 (intellectual strengths), 0.68 and 0.73 (interpersonal strengths), and 0.75 and 0.76 (strengths of restraint). Additionally, we calculated item intercorrelations and corrected item-total correlations. Items showed good internal consistencies for all factors (see Supplementary Material II^1^).

The Spanish translation of the *12-item General Health Questionnaire* (*GHQ-12*; Goldberg and Williams, 1988) was used to assess mental health. The 12-item general health questionnaire is a widely used screening instrument for common mental disorders, and it is used as a general measure of mental health. It measures aspects such as depression, anxiety, social functioning, and loss of confidence. Specifically, the 12 items measure whether a person is able to concentrate, whether they are losing sleep over worry, whether a person feels that they are playing a useful part in life, feels capable of making decisions, feels constantly under strain, feels that they are unable to overcome difficulties, are able to enjoy day-to-day activities, are able to face problems, are feeling unhappy and depressed, are losing confidence, are thinking of themselves as worthless, and are feeling reasonably happy (Sánchez-López and Dresch, 2008). The items assess the severity of these mental problems over recent weeks on a 4-point Likert-type scale (0 to 3). Higher scores indicate worse mental health. In this study, we modified the instructions and asked participants to rate the items considering their experience over the past week. In the present study, Cronbach’s alphas were 0.84 at Time 1 and 0.87 at Time 2.

The *Satisfaction with Life Scale* (*SWLS*; Diener et al., 1985) was used to assess life satisfaction, i.e., the cognitive component of subjective well-being. It is a 5-item questionnaire for the subjective assessment of global life satisfaction in a 7-point answer format (from 1 = strongly disagree to 7 = strongly agree). Higher scores reflect higher life satisfaction. We used the Spanish version (Vázquez et al., 2013). A sample item is: “I am satisfied with my life.” Cronbach alphas in the present study were 0.86 at Time 1 and 0.87 at Time 2.

The *Scale of Positive and Negative Experience* (*SPANE*; Diener et al., 2010) was used to measure positive and negative affect, i.e., the affective component of subjective well-being. The scale measures subjective feelings of well-being (6 items) and ill-being (6 items). In the current study, we measured affect as state since we asked participants to rate their feelings over the past week. The Spanish version was used (Daniel-González et al., 2019). Responses range from 1 (very rarely or never) to 5 (very often or always). Higher scores in these two subscales represent higher positive affect and higher negative affect, respectively. Cronbach alphas in the present study were, for positive affect, 0.92 at Time 1 and 0.93 at Time 2, and for negative affect, 0.86 at both Time 1 and Time 2.

### Procedure

This study has a longitudinal design with two measurement moments: Time 1 and Time 2. Participants were recruited through a message that included an invitation to voluntarily participate in the study sent to their mobile phone or by email with the snowball sampling method. Firstly, we sent the invitation to participate in the study with a link to the online survey to acquaintances, friends, and family by mobile phone (i.e., WhatsApp) and asked them to spread this invitation to their contacts. Simultaneously, we sent the same invitation via email to all members of our university and asked them to spread the study. Lastly, the same invitation was posted on Twitter by one of the study’s coauthors. In this invitation, potential participants were informed of the study’s goals and their rights as research participants, and they were asked for their voluntary participation by completing an online questionnaire (Time 1). Participants who agreed signed an informed consent and completed the questionnaire at Time 1. At the end of the Time 1 questionnaire, they were asked whether they could be contacted in the future. Participants who agreed wrote their email in a blank space in the survey and were contacted again via email to answer the questionnaire at Time 2.

Time 1 data collection was conducted between March 21, 2020 and April 2, 2020, i.e., approximately the second week of lockdown in Spain. Time 2 data collection was conducted between April 24, 2020, and May 18, 2020, right after the Spanish government announced its intention to progressively release the lockdown. The average number of days between Time 1 and Time 2 across participants was 35.53 days (*SD* = 5.97). Participants completed online measures of sociodemographic data, information regarding their situation in relation to COVID-19, character strengths, general mental health, and subjective well-being (i.e., life satisfaction, positive affect and negative affect). Sociodemographic data and most COVID-related information were only measured at Time 1. All remaining variables were measured at both measurement times, i.e., Time 1 and Time 2. Although online data collection has been criticized (e.g., for possible sample biases), empirical evidence shows that data obtained online are comparable to data collected in more conventional ways (e.g., Gosling et al., 2004). The study complied with the University’s ethical standards.

## Results

### Data Reduction

Following the same procedure as previous related research (e.g., Ruch et al., 2010; Martínez-Martí and Ruch, 2017), a principal component analysis with the 24 character strengths was conducted with character strength scores at Time 1 and also at Time 2. Five factors were extracted (Promax rotation). At Time 1 these five factors accounted for 59.59% of the variance. The first 10 eigenvalues were 8.49, 1.92, 1.47, 1.39, 1.01, 0.97, 0.87, 0.81, 0.67, and 0.64. At Time 2 these five factors accounted for 61.72% of the variance. The first 10 eigenvalues were 9.06, 1.94, 1.44, 1.33, 1.02, 0.96, 0.87, 0.74, 0.66, and 0.65. The factor loadings of the 24 character strengths in these five factors at both Time 1 and Time 2 are shown in Table 2.

**TABLE 2:** Factor loadings (pattern matrix) of the 24 character strengths on the five factors in Times 1 and 2 (*N* = 348).

	Time 1					Time 2				
		
	1	2	3	4	5	1	2	3	4	5
Spirituality	**0.73**	0.03	–0.32	0.13	–0.04	0.08	**0.62**	–0.22	0.08	0.00
Bravery	**0.72**	0.04	0.14	–0.19	0.05	–0.01	**0.74**	0.23	–0.12	–0.03
Persistence	**0.65**	–0.03	0.03	0.16	–0.06	–0.01	**0.66**	0.16	0.23	–0.33
Hope	**0.64**	0.19	–0.09	0.04	0.15	0.18	**0.50**	–0.07	0.05	0.29
Leadership	**0.62**	–0.22	0.14	–0.09	0.45	–0.26	**0.83**	0.00	0.01	0.22
Vitality	**0.60**	0.26	0.01	–0.05	0.13	0.15	**0.76**	–0.01	–0.09	0.03
Kindness	–0.04	**0.85**	–0.06	–0.06	0.14	**0.87**	–0.11	0.06	–0.08	0.09
Love	0.05	**0.80**	0.07	–0.16	0.13	**0.86**	–0.04	0.06	–0.19	0.13
Gratitude	0.18	**0.65**	0.11	0.07	–0.22	**0.61**	0.02	0.19	0.09	0.01
Forgiveness	0.11	**0.43**	–0.22	0.32	0.18	**0.56**	0.15	–0.23	0.17	0.12
Integrity	–0.13	**0.40**	0.33	0.26	0.04	**0.51**	0.20	0.30	0.04	–0.26
Curiosity	–0.14	–0.02	**0.88**	–0.04	0.02	0.00	–0.10	**0.82**	–0.13	0.25
Love learning	–0.03	0.14	**0.82**	–0.05	–0.06	0.08	0.20	**0.75**	–0.04	–0.17
Open-minded	–0.22	–0.19	**0.65**	0.49	0.15	–0.09	–0.29	**0.70**	0.46	0.18
Creativity	0.18	0.03	**0.59**	–0.27	0.25	0.03	0.13	0.42	–0.26	**0.54**
Perspective	0.31	–0.17	**0.39**	0.17	0.24	–0.07	0.16	**0.41**	0.23	0.32
Prudence	–0.05	–0.02	–0.06	**0.90**	–0.02	0.14	–0.12	0.01	**0.84**	–0.13
Self-regulation	0.44	–0.31	–0.04	**0.69**	–0.09	–0.32	0.20	0.04	**0.85**	0.08
Humility	–0.01	0.23	–0.05	**0.61**	0.09	0.51	–0.11	–0.06	**0.51**	–0.06
Fairness	–0.02	0.19	0.08	**0.49**	0.07	0.14	0.06	–0.01	**0.51**	0.04
Social intelligence	0.09	0.26	0.03	0.22	**0.51**	**0.40**	0.06	0.01	0.14	0.35
Humor	0.05	0.21	0.23	–0.07	**0.48**	0.15	–0.06	0.12	0.01	**0.75**
Citizenship	–0.06	0.46	–0.09	0.21	**0.47**	0.35	0.22	–0.16	0.17	**0.37**
Apprecbeauty	0.32	0.20	**0.37**	0.16	–0.45	0.22	0.05	**0.46**	0.04	0.05

*Highest factor loadings of each character strength in bold. Apprecbeauty, appreciation of beauty and excellence.*

The character strength factor structures were highly consistent across the two time measurement points (i.e., at Time 1 and at Time 2), with some small inconsistencies. Specifically, creativity and social intelligence loaded different factors at T2 (see Table 2). In order to keep consistency in the content of the character strength factors across Time 1 and Time 2, and after a careful examination of the factor loadings of each strength at Times 1 and 2, we decided to compute five character strength factors that would be equivalent at T1 and T2. We interpreted the first factor as *fortitude strengths*, and included spirituality, bravery, persistence, hope, leadership, and vitality. We took the second factor to be *goodness strengths*, and included kindness, love, gratitude, forgiveness, and integrity. We interpreted the third factor as *intellectual strengths*, and included curiosity, love of learning, open-mindedness, creativity, perspective, and appreciation of beauty and excellence. A fourth factor was interpreted as *strengths of restraint*, and included prudence, self-regulation, humility, and fairness. We interpreted the fifth and final factor as *interpersonal strengths*, and included humor, citizenship, and social intelligence. The mean scores of the character strengths included in each factor were used for subsequent analyses.

### Descriptive Statistics

Table 3 presents the descriptive statistics of the measures of the study.

**TABLE 3:** Descriptive statistics (*N* = 348).

Descriptive statistics						

	M	SD	Range		Skewness	Kurtosis
			
			Actual	Potential		
Fortitude Strengths Time 1	6.17	1.39	1.50–8.83	1–9	–0.48	–0.01
Goodness Strengths Time 1	7.32	1.04	2.40–9.00	1–9	–0.81	1.57
Intellectual Strengths Time 1	6.87	1.10	1.83–9.00	1–9	–0.72	1.40
Restraint Strengths Time 1	6.45	1.30	1.50–9.00	1–9	–0.56	0.37
Interpersonal Strengths Time 1	7.04	1.19	2.33–9.00	1–9	–0.75	0.96
Mental Health Time 1	1.09	0.47	0.25–2.67	0–3	0.75	0.20
Life satisfaction Time 1	3.54	0.82	1–5	1–7	–0.49	–0.18
Positive affect Time 1	3.31	0.76	1–5	1–5	–0.19	–0.03
Negative affect Time 1	2.84	0.88	1–5	1–5	0.03	–0.79
Mental Health Time 2	2.58	1.14	0.08–2.58	0–3	0.64	–0.07
Life satisfaction Time 2	3.58	0.82	1–5	1–7	–0.53	–0.22
Positive affect Time 2	3.45	0.76	1–5	1–5	–0.41	0.15
Negative affect Time 2	2.67	0.85	1–4.83	1–5	0.05	–0.55

### Intercorrelations Among the Variables of the Study

We tested the relationships between character strength factors at Time 1, and mental health, life satisfaction, and positive and negative affect at both Time 1 and Time 2 (see Table 4). The correlations of the 24 individual character strengths at Time 1 with mental health, life satisfaction, and positive and negative affect at Times 1 and 2 are shown in Supplementary Material I (see text footnote 1).

**TABLE 4:** Correlations among the variables of the study (*N* = 348).

	Time 1				Time 2			
		
Strengths Time 1	MH	LS	PA	NA	MH	LS	PA	NA
Fortitude	−0.32**	0.44**	0.38**	−0.33**	−0.30**	0.44**	0.37**	−0.29**
Goodness	–0.09	0.31**	0.23**	−0.14**	−0.17**	0.27**	0.28**	−0.16**
Intellectual	−0.21**	0.28**	0.30**	−0.12*	−0.20**	0.30**	0.34**	−0.13*
Restraint	−0.14**	0.26**	0.18**	−0.19**	−0.18**	0.25**	0.19**	−0.21**
Interpersonal	−0.21**	0.30**	0.31**	−0.24**	−0.26**	0.31**	0.34**	−0.25**

MH, mental health; LS, life satisfaction; PA, positive affect; NA, negative affect. *p < 0.05 and **p < 0.01.

### Regression Analyses

In order to test whether character strength factors predicted changes in mental health and subjective well-being (i.e., life satisfaction, positive affect and negative affect) over a period of approximately 1 month, we conducted a series of regression analyses with each character strength factor at Time 1 as a predictor, and mental health, life satisfaction, positive affect and negative affect at Time 2 as dependent variables. Additionally, dependent variables’ baseline levels, i.e., at Time 1, were controlled. Moreover, to test the directionality of the relationship between character strengths and the dependent variables, i.e., to confirm that character strengths predict mental health and subjective well-being over time, but not the other way round, all analyses were reversed. Specifically, the same regression analyses were performed but with mental health, life satisfaction, positive affect, and negative affect at Time 1 as predictors, and character strengths at T2 as dependent variables, controlling for character strengths at Time 1. In the following subsections, results are reported for each dependent variable. Multicollinearity diagnostics were well within acceptable limits in all analyses.

#### Mental Health

Mental health at Time 2 was predicted by all character strengths factors at Time 1 when controlling for mental health at Time 1. After controlling for mental health at Time 1, *fortitude strengths* predicted an additional 1.8% of the variance in mental health at Time 2, *F*_*Change*_ (1,345) = 9.16, *p* = 0.003; *interpersonal strengths* predicted an additional 2.1% of the variance in mental health at Time 2, *F*_*Change*_ (1,345) = 10.41, *p* = 0.001; *strengths of restraint* predicted an additional 1% of the variance in mental health at Time 2, *F*_*Change*_ (1,345) = 5.08, *p* = 0.025; *intellectual strengths* predicted an additional 0.8% of the variance in mental health at Time 2, *F*_*Change*_ (1,345) = 4.20, *p* = 0.041; *goodness strengths* predicted an additional 1.3% of the variance in mental health at Time 2, *F*_*Change*_ (1,345) = 6.43, *p* = 0.012. The statistically significant results of the regression analyses are presented in Table 5. The results of the reversed analyses were not statistically significant.

**TABLE 5:** Regression analyses predicting mental health at Time 2 (*N* = 348).

						Collinearity	
	
	*B*	*SE*	β	*t*	*p*	Tolerance	VIF
Mental Health T1	0.53	0.05	0.50	10.55	0.000	0.90	1.11
Fortitude Strengths T1	–0.62	0.20	–0.14	–3.03	0.003	0.90	1.11

Mental Health T1	0.55	0.05	0.51	11.22	0.000	0.96	1.05
Interpersonal Strengths T1	–0.75	0.23	–0.15	–3.23	0.001	0.96	1.05

Mental Health T1	0.56	0.05	0.53	11.64	0.000	0.98	1.02
Restraint Strengths T1	–0.48	0.21	–0.10	–2.25	0.025	0.98	1.02

Mental Health T1	0.56	0.05	0.52	11.37	0.000	0.96	1.05
Intellectual Strengths T1	–0.52	0.25	–0.09	–2.05	0.041	0.96	1.05

Mental Health T1	0.57	0.05	0.53	11.81	0.000	0.99	1.01
Goodness Strengths T1	–0.67	0.26	–0.11	–2.54	0.012	0.99	1.01

*Coefficients are for each character strengths factor separately.*

#### Life Satisfaction

Life satisfaction at Time 2 was predicted by *fortitude strengths*, *intellectual strengths* and *interpersonal strengths* at Time 1 when controlling for life satisfaction at Time 1. After controlling for life satisfaction at Time 1, *fortitude strengths* predicted an additional 1.5% of the variance in life satisfaction at Time 2, *F*_*Change*_ (1,345) = 11.86, *p* = 0.001; *interpersonal strengths* predicted an additional 0.8% of the variance in life satisfaction at Time 2, *F*_*Change*_ (1,345) = 6.16, *p* = 0.013; *intellectual strengths* predicted an additional 1% of the variance in life satisfaction at Time 2, *F*_*Change*_ (1,345) = 7.70, *p* = 0.006. The results of the regression analyses are presented in Table 6. The results of the reversed analyses were not statistically significant.

**TABLE 6:** Regression analyses predicting life satisfaction at Time 2 (*N* = 348).

						Collinearity	
						
	*B*	*SE*	β	*t*	*p*	Tolerance	VIF
Life satisfaction T1	0.69	0.04	0.69	17.63	0.000	0.81	1.24
Fortitude Strengths T1	0.40	0.12	0.13	3.44	0.001	0.81	1.24

Life satisfaction T1	0.72	0.04	0.72	19.43	0.000	0.91	1.10
Interpersonal Strengths T1	0.32	0.13	0.09	2.48	0.013	0.91	1.10

Life satisfaction T1	0.72	0.04	0.72	19.58	0.000	0.92	1.08
Intellectual Strengths T1	0.38	0.14	0.10	2.78	0.006	0.92	1.08

Life satisfaction T1	0.73	0.04	0.73	19.92	0.000	0.93	1.07
Restraint Strengths T1	0.18	0.12	0.06	1.53	0.128	0.93	1.07

Life satisfaction T1	0.73	0.04	0.74	19.59	0.000	0.90	1.11
Goodness Strengths T1	0.17	0.15	0.04	1.16	0.248	0.90	1.11

*Coefficients are for each character strengths factor separately.*

#### Positive Affect

Positive affect at Time 2 was predicted by all character strength factors, except the *strengths of restraint* (although there was a tendency: *p* = 0.08), at Time 1 when controlling for positive affect at Time 1. After controlling for positive affect at Time 1, *fortitude strengths* predicted an additional 1.9% of the variance in positive affect at Time 2, *F*_*Change*_ (1,345) = 11.22, *p* = 0.001; *interpersonal strengths* predicted an additional 2.5% of the variance in positive affect at Time 2, *F*_*Change*_ (1,345) = 14.58, *p* < 0.001; *intellectual strengths* predicted an additional 2.6% of the variance in positive affect at Time 2, *F*_*Change*_ (1,345) = 15.37, *p* < 0.001; *goodness strengths* predicted an additional 1.9% of the variance in positive affect at Time 2, *F*_*Change*_ (1,345) = 11.32, *p* = 0.001. The results of the regression analyses are presented in Table 7. The results of the reversed analyses were not statistically significant.

**TABLE 7:** Regression analyses predicting positive affect at Time 2 (*N* = 348).

						Collinearity	
						
	*B*	*SE*	β	*t*	*p*	Tolerance	VIF
Positive affect T1	0.56	0.04	0.57	12.65	0.000	0.85	1.17
Fortitude Strengths T1	0.49	0.15	0.15	3.35	0.001	0.85	1.17

Positive affect T1	0.57	0.04	0.57	13.27	0.000	0.91	1.10
Interpersonal Strengths T1	0.63	0.17	0.17	3.82	0.000	0.91	1.10

Positive affect T1	0.57	0.04	0.57	13.34	0.000	0.91	1.10
Intellectual Strengths T1	0.70	0.18	0.17	3.92	0.000	0.91	1.10

Positive affect T1	0.59	0.04	0.59	13.90	0.000	0.95	1.06
Goodness Strengths T1	0.63	0.19	0.14	3.36	0.001	0.95	1.06

Positive affect T1	0.61	0.04	0.61	14.36	0.000	0.97	1.03
Restraint Strengths T1	0.26	0.15	0.07	1.74	0.082	0.97	1.03

*Coefficients are for each character strengths factor separately.*

#### Negative Affect

Negative affect at Time 2 was predicted by *fortitude strengths*, *strengths of restraint*, and *interpersonal strengths* (in strengths of goodness there was a tendency: *p* = 0.07), at Time 1 when controlling for negative affect at Time 1. After controlling for negative affect at Time 1, *fortitude strengths* predicted an additional 1.1% of the variance in negative affect at Time 2, *F*_*Change*_ (1,345) = 5.93, *p* = 0.015; *interpersonal strengths* predicted an additional 1.3% of the variance in negative affect at Time 2, *F*_*Change*_ (1,345) = 6.86, *p* = 0.009; *strengths of restraint* predicted an additional 0.8% of the variance in negative affect at Time 2, *F*_*Change*_ (1,345) = 4.47, *p* = 0.035. The results of the regression analyses are presented in Table 8. The results of the reversed analyses were not statistically significant.

**TABLE 8:** Regression analyses predicting negative affect at Time 2 (*N* = 348).

						Collinearity	
						
	*B*	*SE*	β	*t*	*p*	Tolerance	VIF
Negative affect T1	0.54	0.04	0.56	12.31	0.000	0.89	1.12
Fortitude Strengths T1	–0.41	0.17	–0.11	–2.44	0.015	0.89	1.12

Negative affect T1	0.55	0.04	0.57	12.86	0.000	0.94	1.06
Interpersonal Strengths T1	–0.50	0.19	–0.12	–2.62	0.009	0.94	1.06

Negative affect T1	0.56	0.04	0.58	13.16	0.000	0.96	1.04
Strengths of Restraint T1	–0.37	0.17	–0.09	–2.12	0.035	0.96	1.04

Negative affect T1	0.57	0.04	0.59	13.51	0.000	0.99	1.02
Intellectual Strengths T1	–0.29	0.20	–0.06	–1.45	0.148	0.99	1.02

Negative affect T1	0.56	0.04	0.58	13.41	0.000	0.98	1.02
Goodness Strengths T1	–0.38	0.21	–0.07	–1.76	0.079	0.98	1.02

*Coefficients are for each character strengths factor separately.*

## Discussion

This study provides original evidence on the positive association between character strengths and resilience (operationalized as stable or increased mental health and well-being despite an adverse situation) over a specific period during the COVID-19 pandemic in Spain. What is more, this study shows that, overall, character strengths predicted an *increase* in mental health and subjective well-being, which, although small, we believe is relevant considering the current adverse circumstances. Specifically, all character strength factors (i.e., *fortitude strengths*, *goodness strengths*, *intellectual strengths*, *strengths of restraint*, and *interpersonal strengths*) predicted an increase in mental health, and an increase in positive affect, with the exception of *strengths of restraint*. *Fortitude strengths* (i.e., spirituality, bravery, persistence, hope, leadership, and vitality), *intellectual strengths* (i.e., curiosity, love of learning, open-mindedness, creativity, perspective, and appreciation of beauty and excellence), and *interpersonal strengths* (i.e., humor, citizenship, and social intelligence) predicted an increase in life satisfaction. Finally, *fortitude strengths*, *interpersonal strengths*, and *strengths of restraint* (i.e., prudence, self-regulation, humility, and fairness), predicted a decrease in negative affect.

Moreover, none of the reversed analyses yielded significant effects. This means that mental health and subjective well-being did not predict changes in character strengths over a period of approximately 1 month, a result that further supports the directionality of the relationship between character strengths and mental health and subjective well-being. Nonetheless, we must limit this interpretation to the length of the period studied, i.e., approximately 1 month, and to the current situation. The results observed do not exclude the possibility that well-being and mental health could change character strengths over longer periods or under other circumstances. Future longer longitudinal studies will be helpful to examine this issue. Therefore, in general, the hypothesis of the study was met.

The five individual character strengths (at Time 1) with the highest correlations with mental health (at Time 2) were hope, vitality, self-regulation, social intelligence, and humor. The five individual character strengths (at Time 1) with the highest correlations with life satisfaction (at Time 2) were vitality, hope, bravery, persistence, and self-regulation. The five individual character strengths (at Time 1) with the highest correlations with positive affect (at Time 2) were hope, vitality, creativity, social intelligence, and curiosity. Finally, the five individual character strengths (at Time 1) with the highest correlations with negative affect (at Time 2) were hope, vitality, self-regulation, social intelligence, and bravery. These results are generally in line with previous research on the relationship between character strengths and well-being and resilience (e.g., Peterson et al., 2007; Littman-Ovadia and Lavy, 2012; Azañedo et al., 2014; Martínez-Martí and Ruch, 2014, 2017). While hope and vitality seem to be the character strengths with the highest correlations with all the mental health and subjective well-being indicators in this study, a finding replicated repeatedly in previous research on character strengths under less adverse circumstances, what is new in this context is the relevance that bravery, social intelligence and self-regulation show in relation to well-being, especially bravery. Bravery is not usually one of the character strengths that shows the highest correlation with negative affect and life satisfaction, but in this adverse context, it seems to be more important for these components of subjective well-being than other character strengths. Likewise, self-regulation and social intelligence seem to be more important for well-being and mental health, relative to other character strengths, in this specific context.

Character strengths were grouped into five factors after conducting a principal component analysis at Time 1 and Time 2, and carefully examining the factor loadings of all character strengths in the five factors at both measurement times. The factor structure observed was highly consistent across Time 1 and Time 2, but slightly different from the factor structures observed in previous studies (e.g., Ruch et al., 2010; Martínez-Martí and Ruch, 2017). These factors are slightly different from the factors reported in Ruch et al. (2014), although most of the content overlaps. Ruch et al. (2014) labeled the five factors as *interpersonal strengths* (i.e., love, kindness, social intelligence, citizenship, fairness, leadership, forgiveness, and humor), *intellectual strengths* (i.e., creativity, curiosity, open-mindedness, love of learning, perspective, and appreciation of beauty and excellence), *emotional strengths* (i.e., bravery, persistence, vitality, and hope), *strengths of restraint* (i.e., honesty, humility, prudence, and self-regulation) and *theological strengths* (i.e., gratitude and spirituality). However, except where there were differences, the resulting factors in this study were easily interpretable and distinct from each other from a conceptual point of view.

We labeled the first factor to emerge *fortitude strengths*, which is particularly interesting. This factor systematically yielded the highest correlations with all the variables in the study: mental health and subjective well-being. Its configuration was somewhat new as it grouped all character strengths that, at first sight, could be associated with a strong and resilient person: spirituality, bravery, persistence, hope, leadership, and vitality (in decreasing order of factor loadings). The originality of this factor lies in the combination of stamina, associated with the virtue of courage, with the capacity to transcend the (distress of the) current situation, which relates to the virtue of transcendence.

What is particularly striking is that spirituality has the highest loading on that factor, when normally spirituality is grouped with gratitude, or appreciation of beauty and excellence (e.g., Martínez-Martí and Ruch, 2017), i.e., with other transcendence strengths, but not with courage strengths. Spirituality emerges as a driving force, grouping almost all the character strengths pertaining to the virtue of courage, i.e., bravery, vitality and persistence, plus the character strengths of hope and leadership. In the present study, spirituality was assessed as having coherent beliefs about the higher purpose and meaning of life, which might include religious beliefs, but it has a broader scope. Spirituality has been linked to positive mental and physical health functioning (Nooney and Woodrum, 2002; Powell et al., 2003). Additionally, spirituality might be especially helpful when people experience adverse events. After the terrorist attacks in New York on September 11, 2001, more than 90% of the people interviewed reported that they coped by “turning to religion,” second only to “talking with others” (Schuster et al., 2001). Spirituality might offer a positive meaning-making framework for coping (Park, 2005) in the current pandemic, and enhance both social support, despite the isolation, and effective cognitive processing of this stressful event (McIntosh et al., 1993).

On the other hand, leadership involves encouraging a group (of which one is a member) to get things done, while at the same time maintaining good relations within the group and treating everyone equally. This might have been particularly important in the current situation, as all pre-established routines at work and at home have been disrupted and a reorganization of all daily tasks has had to be done. Hope and vitality, two of the other character strengths that belong to the fortitude strengths factor, have already shown their importance in terms of well-being and resilience in cross-sectional research on character strengths (e.g., Martínez-Martí and Ruch, 2014, 2017). Vitality provides energy and enthusiasm, while hope provides a positive outlook of the future that keeps the motivation to keep going high, which in this uncertain situation might be vital. Finally, the presence of bravery and persistence (together with vitality) in the *fortitude strengths* factor highlights the importance of the virtue of courage for resilience. Some authors (e.g., Maddi, 2004; Jordan, 2005; Martínez-Martí and Ruch, 2017) have previously suggested that resilience involves courage. The results of this study support that claim and provide novel evidence reflecting that courage combined with transcendence seem to help people navigate the current pandemic with better mental health and well-being.

The second factor, which we labeled *goodness strengths*, grouped kindness, love, gratitude, forgiveness, and integrity. This factor included character strengths pertaining to the virtues of humanity (all except social intelligence), transcendence, temperance, and courage. They are somehow interpersonal too, but their focus is more on the human quality of a kind-hearted human being. This factor also predicted mental health and positive affect. Many of these character strengths are directly related to positive emotional states (e.g., love, gratitude) and all facilitate positive relationships. In this lockdown, where other people in the same household might be a source of support at times but also a source of potential tension, character strengths such as kindness, forgiveness, love and gratitude might be particularly helpful.

The third factor, which we labeled *intellectual strengths*, included curiosity, love of learning, open-mindedness, creativity, perspective and appreciation of beauty and excellence, i.e., all the character strengths pertaining to the virtue of wisdom plus appreciation of beauty and excellence, which is sometimes grouped with intellectual strengths (e.g., Martínez-Martí and Ruch, 2017), even though it belongs to the virtue of transcendence. This factor predicted mental health, life satisfaction, and positive affect. The relevance of *intellectual strengths* in the current situation may be due to the strong requirements of having to adapt to a new way of life. Strengths such as curiosity, love of learning, open-mindedness, creativity, perspective and appreciation of beauty and excellence, could facilitate a better adaptation to the demands of the environment. During this lockdown period, the population has needed to learn different ways of working, studying, relaxing and getting along with their nearest and dearest, among other daily habits. For example, a significant percentage of the population who carried on working or studying from home needed to learn or improve their e-skills; some people had to develop different ways of achieving their professional goals in a remote work environment; others maybe saw this period as an opportunity for spending time on their own and with their relatives and/or improving their professional profile. Specifically, 70% of the sample carried on working (60% worked from home), so it is possible that many participants needed to react quickly to deal with professional circumstances as soon as they went into lockdown. In this sense, *intellectual strengths* foster the exploration of situational conditions and the production of new strategies for problem solving (Peterson and Seligman, 2004), and seem to be linked to coping with stress in the work environment (Harzer and Ruch, 2015). Successfully adapting to new environments could have facilitated a better management of stress during this confinement period, and positively affected mental health and subjective well-being. Under such conditions *intellectual strengths* may therefore provide better skills to look for new and creative responses to tackle the changes required.

A fourth factor, which we labeled *strengths of restraint*, included prudence, self-regulation, and humility (all character strengths pertaining to the virtue of temperance), and fairness (which pertains to the virtue of justice). Although *strengths of restraint* did not predict positive affect or life satisfaction, they predicted better mental health and a decrease in negative affect. Prudence and self-regulation are character strengths that act as moderators of behavior and emotions (Peterson and Seligman, 2004). The benefits of this character strength factor in the current situation may lie mainly in its ability to buffer the stress response. The COVID-19 pandemic represents a major stressor. This pandemic enforced a global lockdown for personal and common good, but to the detriment of individual freedom. These enforced restrictions (e.g., being confined at home, following all the safety requirements when going outside) might be very stressful for individuals, especially for those individuals who are low in strengths of restraint, because these restrictions demand a high level of self-control. The response to stress from a transactional point of view (Lazarus and Folkman, 1984) depends on the stressor appraisal processes and the coping resources available to cope with it. Strengths of restraint may facilitate coping with stress in several ways. Self-regulation and prudence might facilitate an adequate reappraisal of the perceived risk and the coping resources available to deal with it (maybe through the perception that one can comply with the lockdown requirements by means of self-control), thus minimizing the perception of threat and consequently, negative affect (i.e., worry, fear, anxiety, or panic).

Additionally, prudence enables people to consider the consequences of their actions, which might facilitate the fulfillment of the preventative measures (i.e., social distancing, confinement) taken to reduce the spread of infection. In this sense, prudence and self-regulation might support effective self-management and a sense of controlling the situation. In fact, *strengths of restraint* have shown moderate positive correlations with self-efficacy (Martínez-Martí and Ruch, 2017). In a similar way, fairness and humility may promote a parallel self-control, but applied at a community level. The current situation demands an equal distribution of resources among the members of the community and the prioritization of these resources to the people who need them most, so as to avoid the collapse of the health services, the depletion of resources or the hoarding of protective items such as face masks, hydroalcoholic gel, food, and supplies. While for some people this may constitute a source of stress, people with high levels of fairness and humility might fulfill these community requests more easily and, thus, experience less distress.

Overall, the present results suggest that people with great *strengths of restraint* might have adapted more easily to this enforced restrictions, as they were already more capable of restraining their own emotions and behaviors for the sake of preserving their own well-being and the well-being of others. Strengths of restraint seem to fit the definition of well-being adopted by Dodge et al. (2012), i.e., the balance point between an individual’s resource pool and the challenges faced.

Finally, we labeled the fifth factor *interpersonal strengths*, which included humor, social intelligence and citizenship. This factor predicted an increase in mental health, life satisfaction, positive affect, and a decrease in negative affect. These character strengths might have played a significant role in releasing tension, fostering social connectedness and support, and a sense of community in this period of isolation. In fact, previous research has shown that these character strengths yielded positive moderate correlations with social support and resilience (Martínez-Martí and Ruch, 2017).

The pandemic has changed the way people perceive and relate to each other (Rosa et al., 2020). It is possible that people high in *interpersonal strengths*, especially in social intelligence, have adapted better to this new way of relating to others. Additionally, people with great *interpersonal strengths*, particularly citizenship, might have been more involved in collective civic rituals that facilitate collective coping, and benefit more from them. For example, throughout lockdown, every day at 8 pm, people would go onto their balconies and clap for a few minutes to express their support for all the professionals in Spain actively working to look after the population and ensure that society as a whole functioned adequately during the pandemic.

Meanwhile, humor seems to facilitate adaptive coping with stress, enhance social interactions and well-being, and decrease stress and negative emotions (Kuiper, 2012; Ruch and Hofmann, 2017). During the first days of lockdown in Spain, there was an explosion of jokes regarding the ways people would adapt to lockdown. This example shows how the use of humor probably helped people share and release the distress caused by the severe restrictions imposed by the government. In the current context, *interpersonal strengths* may have acted as a protective mechanism against the fatalities of the health crisis and as a social lubricant for the new social contexts.

This study has several limitations that should be considered when interpreting the results. Firstly, we used a convenience sample, which is not representative of the general Spanish population. In fact, the sample was composed mainly of women and highly educated participants. Moreover, given that participation was voluntary, it is possible that people who are more extrovert, prosocial and resilient were the ones that decided to participate in the study. In this sense, the data might be biased. Secondly, we analyzed the potential protective role of character strengths in a Spanish sample, but it is possible that the way character strengths influence mental health and well-being in this particular situation vary in other countries. Therefore, it is possible that our results cannot be generalized for other countries. We believe cultural and societal factors might influence how people have managed the current situation, so it would be interesting to see if the results observed in this study replicate in other studies conducted in other countries with a different culture. Thirdly, since the data presented are self-reported, data could be biased and should be considered carefully. Future studies should use more objective measures that complement self-reporting measures. Fourthly, the variance explained by character strengths was small. This could be partially explained by the use of a brief instrument to assess character strengths. Because only one item is used to measure each character strength, relationships are usually underestimated. Fifthly, the period between T1 and T2 (approximately 1 month) was very short. Time 1 data collection was conducted after the government announced the lockdown in Spain (approximately the second week of lockdown), and Time 2 data collection was conducted right after the Spanish government announced the intention to progressively release the lockdown. Longitudinal studies usually cover longer periods, but in this study, we tried to capture any possible change during the lockdown, and thus the two measurement points were dictated by the evolution of the lockdown decreed in Spain. Longer longitudinal studies would be necessary to explore how character strengths might help individuals deal with the pandemic in the long term.

This study expands the current theory on the role of character strengths in adverse situations by showing that character strengths might help increase mental health and subjective well-being during the COVID-19 pandemic. These results have some practical implications. We believe preventive character strength interventions in the current pandemic would be beneficial for the general population. Although all character strength factors predicted at least some of the variables in the study (i.e., mental health, life satisfaction, and positive and negative affect), *fortitude strengths* and *interpersonal strengths* yielded the highest correlations. Therefore, we suggest focusing on the development of these two character strength factors, which broadly involve transcending the current situation and the connection with other people by having a particularly positive outlook on the future, approaching the current situation with energy and determination, and relating to others in a conscious, supportive, and positive way.

Within these factors, character strengths such as hope, vitality, humor and social intelligence generally showed the highest correlations with the variables in the study. Hope might be developed by visualizing and writing about the best possible self at some point in the future (Meevissen et al., 2011). In the current situation, we would extend this intervention to visualizing a positive future overall, when the pandemic is over. Also, setting a goal and writing down many pathways to achieving this goal and the reasons why the person will be able to achieve it might foster hopeful thinking (Feldman and Dreher, 2012). Maybe these goals could be related to the current pandemic, e.g., goals related to fostering one’s well-being and the well-being of other people who might be suffering in the current situation. Additionally, we suggest that people set a goal that allows them to use their character strengths, as using character strengths is associated with greater vitality and well-being (Dubreuil et al., 2014). Likewise, setting goals that are aligned with intrinsic values for self-determined reasons would be most beneficial, as pursuing intrinsic values for self-determined reasons has been associated with greater well-being and vitality (Kasser and Ryan, 1993).

Vitality could also be fostered by behaving prosocially (Martela and Ryan, 2016), spending time outside (when possible), especially in nature (Ryan et al., 2010), or by sharing positive events (Lambert et al., 2011). As far as humor and playfulness are concerned, spending time playing with family and friends will help to cultivate a playful attitude and a sense of fun and connection with others (McGhee, 2010). In addition, writing about the funniest things that happened during the day might also foster humor (Wellenzohn et al., 2016). Meanwhile, social intelligence might be nurtured by identifying and labeling emotions as they occur, and by expressing them to others in a balanced way (Nelis et al., 2009), and by practicing mindfulness (Schutte and Malouff, 2011).

To sum up, this longitudinal study provides original evidence showing that character strengths seem to promote resilience over time in adverse situations such as the current pandemic. Based on the results observed, we have offered some possible interpretations about the unique ways in which character strengths might be fostering mental health and well-being. Finally, we recommend the implementation of preventive character strengths interventions, and suggest some specific character strength-based interventions, to preserve mental health during the current pandemic. Future longer longitudinal studies with more representative samples, which allow for a cross-cultural analysis, and with more objective measures would be very valuable. It would be interesting to explore in more detail how individuals apply their strengths to improving their well-being and mental health during the current pandemic, maybe incorporating some qualitative measures as well, and to devise and test character strength-based interventions specifically designed for the current context to help people develop their resilience.

## Data Availability Statement

The dataset is available as Supplementary Material III at (Frontiers’ link) and at https://osf.io/n2sqc/.

## Ethics Statement

Ethical review and approval was not required for the study on human participants in accordance with the local legislation and institutional requirements. The patients/participants provided their written informed consent to participate in this study.

## Author Contributions

MM-M, CT, DP, and GC contributed to the conception and design of the work, data collection, data analysis and interpretation, drafting and critical revision of the article, and final approval of the published version. All authors contributed to the article and approved the submitted version.

## Conflict of Interest

The authors declare that the research was conducted in the absence of any commercial or financial relationships that could be construed as a potential conflict of interest.

## Footnotes

1 https://osf.io/n2sqc/
