# Supplementary material for: Character Strengths Predict an Increase in Mental Health and Subjective Well-Being Over a One-Month Period During the COVID-19 Pandemic Lockdown
Source: Front Psychol. 2020 Oct 21;11:584567. doi: 10.3389/fpsyg.2020.584567 (PMC7609545; doi:10.3389/fpsyg.2020.584567)
Supplement: Supplementary file 2 [file Table_1.docx]

Supplementary material I. *Correlations between the 24 character strengths at Time 1 and mental health, and subjective well-being (Times 1 and 2).*

|  | Time1 | | | | | | | Time 2 | | | | | | | | | |
| --- | --- | --- | --- | --- | --- | --- | --- | --- | --- | --- | --- | --- | --- | --- | --- | --- | --- |
| Strengths Time 1 | MH | LS | | PA | | NA | MH | | LS | | PA | | | NA | |  |  |
| Hope | -.32** | .44** | .39** | | -.36** | | | -.33** | | .40** | | .41** | -.39** | |  |  |  |
| Vitality | -.22** | .39** | .34** | | -.29** | | | -.27** | | .40** | | .36** | -.27** | |  |  |  |
| Self-regulation | -.24** | .33** | .24** | | -.24** | | | -.26** | | .30** | | .24** | -.25** | |  |  |  |
| Social intelligence | -.18** | .29** | .21** | | -.21** | | | -.22** | | .29** | | .31** | -.22** | |  |  |  |
| Humor | -.25** | .21** | .34** | | -.23** | | | -.21** | | .23** | | .28** | -.18** | |  |  |  |
| Perspective | -.15** | .22** | .24** | | -.10 | | | -.21** | | .27** | | .24** | -.14* | |  |  |  |
| Persistence | -.18** | .31** | .26** | | -.15** | | | -.21** | | .33** | | .27** | -.17** | |  |  |  |
| Love | -.12* | .29** | .20** | | -.11* | | | -.20** | | .25** | | .28** | -.18** | |  |  |  |
| Creativity | -.21** | .21** | .30** | | -.12* | | | -.19** | | .23** | | .34** | -.12* | |  |  |  |
| Bravery | -.24** | .34** | .29** | | -.27** | | | -.19** | | .33** | | .22** | -.22** | |  |  |  |
| Spirituality | -.17** | .23** | .20** | | -.18** | | | -.18** | | .23** | | .19** | -.13* | |  |  |  |
| Curiosity | -.18** | .20** | .25** | | -.14* | | | -.17** | | .22** | | .30** | -.15** | |  |  |  |
| Citizenship | -.06 | .20** | .16** | | -.12* | | | -.17** | | .20** | | .20** | -.18** | |  |  |  |
| Leadership | -.28** | .25** | .23** | | -.20** | | | -.16** | | .27** | | .20** | -.16** | |  |  |  |
| Honesty | -.08 | .27** | .15** | | -.13* | | | -.15** | | .27** | | .21** | -.15** | |  |  |  |
| Open-mindedness | -.13* | .18** | .14** | | -.03 | | | -.13* | | .20** | | .20** | -.05 | |  |  |  |
| Apprecbeauty | -.11* | .14* | .11* | | -.05 | | | -.07 | | .12* | | .10 | -.03 | |  |  |  |
| Fairness | -.06 | .20** | .10 | | -.14* | | | -.07 | | .18** | | .07 | -.08 | |  |  |  |
| Forgiveness | -.03 | .19** | .15** | | -.10 | | | -.08 | | .16** | | .15** | -.07 | |  |  |  |
| Humility | -.07 | .16** | .13* | | -.10 | | | -.08 | | .16** | | .12* | -.11* | |  |  |  |
| Gratitude | -.05 | .21** | .15** | | -.09 | | | -.09 | | .19** | | .18** | -.07 | |  |  |  |
| Love of learning | -.09 | .24** | .22** | | -.07 | | | -.10 | | .25** | | .26** | -.08 | |  |  |  |
| Kindness | -.07 | .20** | .21** | | -.11* | | | -.10 | | .15** | | .22** | -.12* | |  |  |  |
| Prudence | -.04 | .09 | .07 | | -.09 | | | -.10 | | .10 | | .11* | -.15** | |  |  |  |
| Note. Apprecbeauty = Appreciation of beauty and excellence. MH = Mental health, LS = Life satisfaction, PA = Positive affect, NA = Negative affect. **p* < .05. ***p* < .01. | | | | | | | | | | | | | | | | |  |
