# Supplementary material for: Character Strengths Predict an Increase in Mental Health and Subjective Well-Being Over a One-Month Period During the COVID-19 Pandemic Lockdown
Source: Front Psychol. 2020 Oct 21;11:584567. doi: 10.3389/fpsyg.2020.584567 (PMC7609545; doi:10.3389/fpsyg.2020.584567)

Supplementary material II. *Reliability Analysis of character strength factors at T1 (pre) and T2 (post).*

| Fortitude PRE Factor Reliability Statistics | | | | | | |
| --- | --- | --- | --- | --- | --- | --- |
|  | | Cronbach's α | | | McDonald's ω | |
| Fortitude PRE |  | 0.804 | |  | 0.828 |  |
| Fortitude PRE Items Reliability Statistics | | | | | | |
|  | | | item-rest correlation | | | |
| Bravery PRE |  | | 0.610 | | |  |
| Spirituality PRE |  | | 0.425 | | |  |
| Hope PRE |  | | 0.641 | | |  |
| Persistence PRE |  | | 0.577 | | |  |
| Leadership PRE |  | | 0.582 | | |  |
| Vitality PRE |  | | 0.665 | | |  |


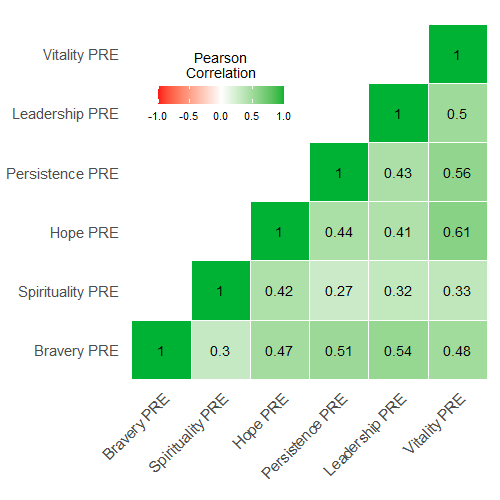


| Goodness PRE Factor Reliability Statistics | | | |
| --- | --- | --- | --- |
|  | | Cronbach's α | McDonald's ω |
| Goodness PRE |  | 0.787 | 0.799 |
| Goodness PRE Item Reliability Statistics | | | |
|  | | item-rest correlation | |
| Kindness PRE | | 0.649 | |
| Love PRE | | 0.631 | |
| Gratitude PRE | | 0.591 | |
| Forgiveness PRE | | 0.481 | |
| Integrity PRE | | 0.518 | |


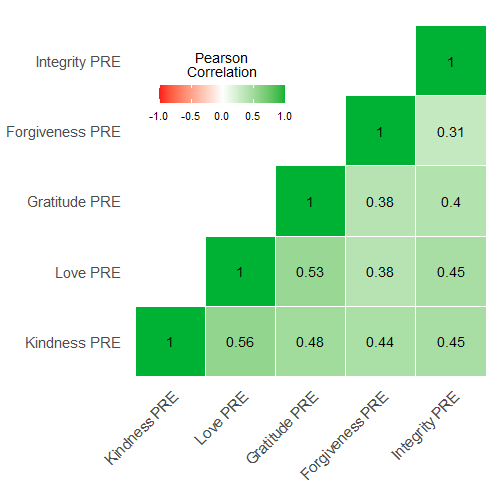


| Intellectual PRE Factor Reliability Statistics | | | | |
| --- | --- | --- | --- | --- |
|  | | Cronbach's α | McDonald's ω | |
| Intellectual PRE |  | 0.795 |  | 0.803 |
| Intellectual PRE Item Reliability Statistics | | | | |
|  | | item-rest correlation | | |
| Curiosity PRE |  | 0.605 |  | |
| Love learning PRE |  | 0.659 |  | |
| Open-minded PRE |  | 0.551 |  | |
| Creativity PRE |  | 0.523 |  | |
| Perspective PRE |  | 0.541 |  | |
| Aprecbeauty PRE |  | 0.440 |  | |


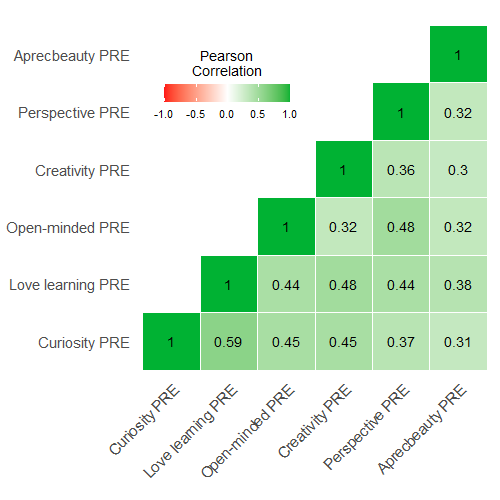


| Restraint PRE Factor Scale Reliability Statistics | | | | | | |
| --- | --- | --- | --- | --- | --- | --- |
|  | | Cronbach's α | | | McDonald's ω | |
| Restraint PRE |  | 0.746 |  | | 0.752 |  |
| Restraint PRE Item Reliability Statistics | | | | | | |
|  | | item-rest correlation | | | | |
| Prudence PRE | | 0.640 | |  | | |
| Self regulation PRE | | 0.542 | |  | | |
| Humility PRE | | 0.564 | |  | | |
| Fairness PRE | | 0.433 | |  | | |


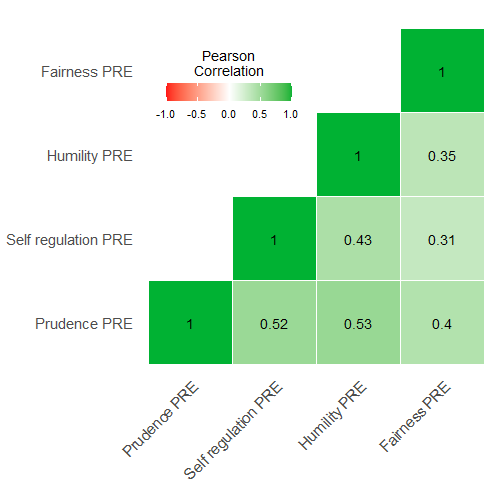


| Interpersonal PRE Factor Scale Reliability Statistics | | |
| --- | --- | --- |
|  | Cronbach's α | McDonald's ω |
| Interpersonal PRE | 0.678 | 0.694 |
| Interpersonal PRE Item Reliability Statistics | | |
|  | item-rest correlation | |
| Social Intelligence PRE | 0.566 | |
| Citizenship PRE | 0.504 | |
| Humor PRE | 0.410 | |


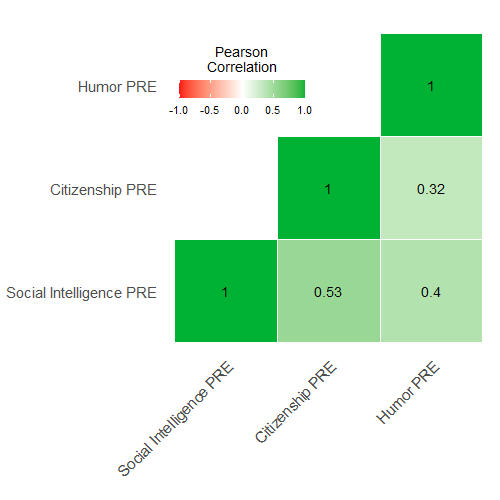


| Fortitude POST Factor Reliability Statistics | | | | | | | | | |  |  |
| --- | --- | --- | --- | --- | --- | --- | --- | --- | --- | --- | --- |
|  | | |  |  |  | | |  |  | |  |
|  | Cronbach's α | | | | | | McDonald's ω | | |  |  |
| Fortitude POST | | | 0.817 | | | | 0.836 | | |  |  |
| Fortitude POST  Item Reliability Statistics | | | | | | | | | |  |  |
|  |  |  | | | |  | | | | | |
|  | item-rest correlation | | | | | | | | |  |  |
| Bravery POST | 0.643 | | | | | | | | |  |  |
| Spirituality POST | 0.450 | | | | | | | | |  |  |
| Hope POST | 0.639 | | | | | | | | |  |  |
| Persistence POST | 0.537 | | | | | | | | |  |  |
| Leadership POST | 0.610 | | | | | | | | |  |  |
| Vitality POST | 0.706 | | | | | | | | |  |  |

 
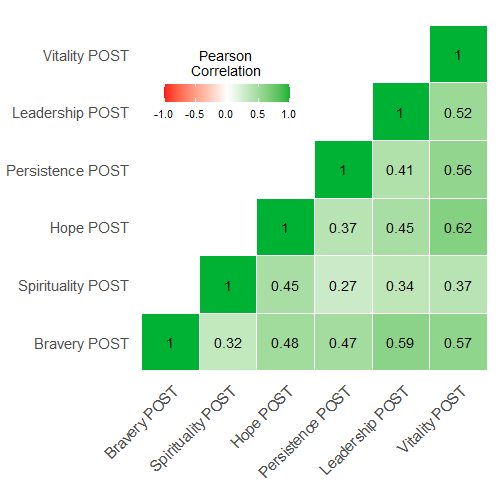


| Goodness POST Factor Reliability Statistics | | | | | |  |  |
| --- | --- | --- | --- | --- | --- | --- | --- |
|  | Cronbach's α | | McDonald's ω | | |  |  |
| Goodness POST |  | 0.814 | |  | 0.824 | | |
| Goodness POST Factor Item Reliability Statistics | | | | | |  |  |
|  | item-rest correlation | | | | | |  |
| Kindness POST | 0.690 | | | | |  |  |
| Love POST | 0.640 | | | | |  |  |
| Gratitude POST | 0.640 | | | | |  |  |
| Forgiveness POST | 0.549 | | | | |  |  |
| Integrity POST | 0.545 | | | | |  |  |

 
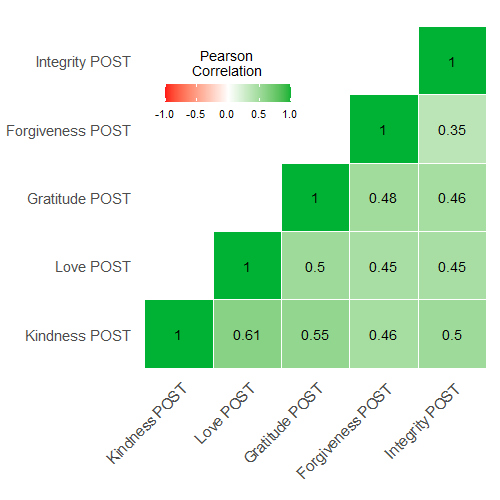


| Intellectual POST Factor Reliability Statistics | | | | | |
| --- | --- | --- | --- | --- | --- |
|  | | | Cronbach's α | | McDonald's ω |
| Intellectual POST |  | | 0.817 |  | 0.826 |
| Intellectual POST Item Reliability Statistics | | | | | |
|  | | | item-rest correlation | | |
| Curiosity POST | |  | 0.669 | | |
| Love learning POST | |  | 0.630 | | |
| Open mindend POST | |  | 0.588 | | |
| Creativity POST | |  | 0.544 | | |
| Perspective POST | |  | 0.612 | | |
| Apreccbeauty POST | |  | 0.489 | | |


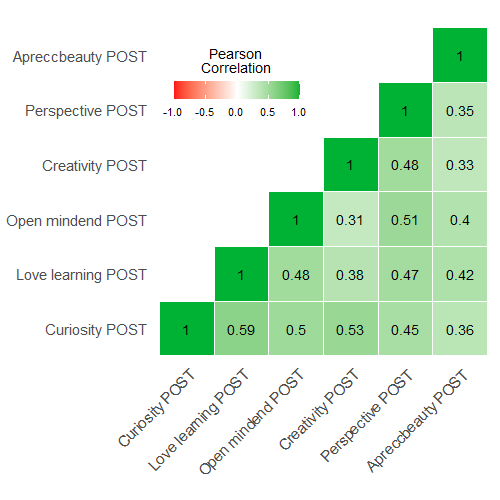


| Restraint POST Factor Reliability Statistics | | | | |  |
| --- | --- | --- | --- | --- | --- |
|  | | Cronbach's α | | McDonald's ω |  |
| Restraint POST |  | 0.760 |  | 0.770 | |
| Restraint Factor POST Reliability Statistics | | | | |  |
|  | | item-rest correlation | | |  |
| Prudence POST | | 0.684 | | |  |
| Self regulation POST | | 0.571 | | |  |
| Humility POST | | 0.575 | | |  |
| Fainess POST | | 0.427 | | |  |


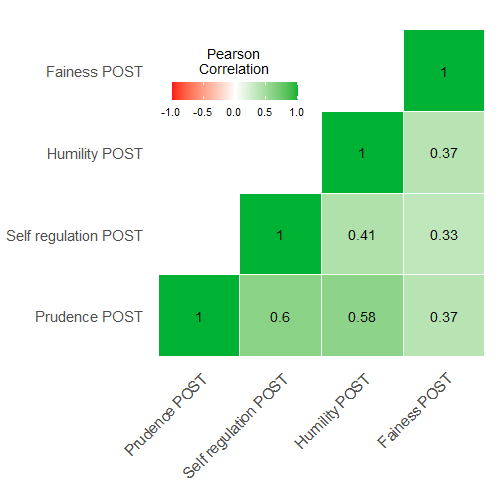


| Interpersonal Factor POST Reliability Statistics | | | | | |
| --- | --- | --- | --- | --- | --- |
|  | | Cronbach's α | | McDonald's ω | |
| Interpersonal POST |  | 0.729 |  | | 0.731 |
| Interpersonal Factor POST Reliability Statistics | | | | | |
|  | | item-rest correlation | | | |
| Humor POST | | 0.522 | | | |
| Social intelligence POST | | 0.544 | | | |
| Citizenship POST | | 0.588 | | | |


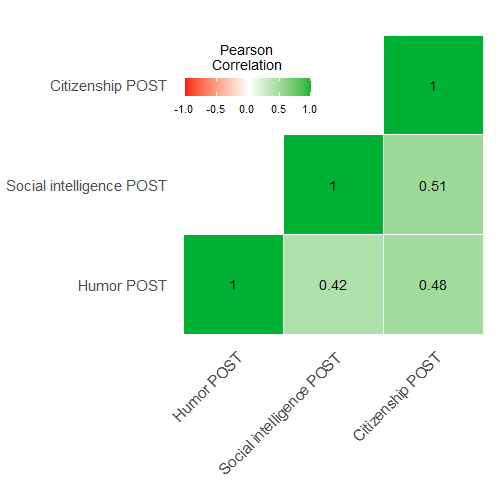

Supplement: Supplementary file 3 [file Table_2.docx]
